# *Bacillus subtilis* as a Platform for Molecular Characterisation of Regulatory Mechanisms of *Enterococcus faecalis* Resistance against Cell Wall Antibiotics

**DOI:** 10.1371/journal.pone.0093169

**Published:** 2014-03-27

**Authors:** Chong Fang, Emanuel Stiegeler, Gregory M. Cook, Thorsten Mascher, Susanne Gebhard

**Affiliations:** 1 Department Biology I, Microbiology, Ludwig-Maximilians-Universität München, Martinsried, Germany; 2 Department of Microbiology and Immunology, Otago School of Medical Sciences, University of Otago, Dunedin, New Zealand; University Medical Center Utrecht, Netherlands

## Abstract

To combat antibiotic resistance of *Enterococcus faecalis*, a better understanding of the molecular mechanisms, particularly of antibiotic detection, signal transduction and gene regulation is needed. Because molecular studies in this bacterium can be challenging, we aimed at exploiting the genetically highly tractable Gram-positive model organism *Bacillus subtilis* as a heterologous host. Two fundamentally different regulators of *E. faecalis* resistance against cell wall antibiotics, the bacitracin sensor BcrR and the vancomycin-sensing two-component system VanS_B_-VanR_B_, were produced in *B. subtilis* and their functions were monitored using target promoters fused to reporter genes (*lacZ* and *luxABCDE*). The bacitracin resistance system BcrR-BcrAB of *E. faecalis* was fully functional in *B. subtilis*, both regarding regulation of *bcrAB* expression and resistance mediated by the transporter BcrAB. Removal of intrinsic bacitracin resistance of *B. subtilis* increased the sensitivity of the system. The *lacZ* and *luxABCDE* reporters were found to both offer sensitive detection of promoter induction on solid media, which is useful for screening of large mutant libraries. The VanS_B_-VanR_B_ system displayed a gradual dose-response behaviour to vancomycin, but only when produced at low levels in the cell. Taken together, our data show that *B. subtilis* is a well-suited host for the molecular characterization of regulatory systems controlling resistance against cell wall active compounds in *E. faecalis*. Importantly, *B. subtilis* facilitates the careful adjustment of expression levels and genetic background required for full functionality of the introduced regulators.

## Introduction

*Enterococcus faecalis* is one of the most common causes of nosocomial infections. Increasing incidences of infections with antibiotic resistant strains, particularly with vancomycin resistant enterococci (VREs), therefore pose a major health risk [1], [2]. Vancomycin is a glycopeptide antibiotic that targets the lipid II cycle of cell wall biosynthesis by binding to the terminal D-alanyl-D-alanine (D-Ala-D-Ala) moiety of peptidoglycan precursors on the surface of the cell, thus inhibiting their incorporation into the cell wall [3]. Many other antimicrobial substances also target the lipid II cycle [4], including bacteriocins and mammalian defensins [5], [6], both of which will likely be encountered by *E. faecalis* in its natural gut habitat. Furthermore, many enterococcal isolates were found to be highly resistant against bacitracin [7], [8], yet another inhibitor of cell wall biosynthesis [9].

The molecular mechanisms leading to resistance are often well known. In the case of vancomycin, high-level resistance is for example ensured by target alteration through replacement of the terminal D-Ala-D-Ala by D-Ala-D-lactate. In VanA-type strains, this is accomplished through the action of the VanHAX system, while in VanB-type strains the VanH_B_BX_B_ proteins mediate resistance [10], [11]. High-level bacitracin resistance of *E. faecalis* is conferred by the ATP-binding cassette (ABC) transporter BcrAB, which presumably removes the antibiotic from its site of action (i.e. the cytoplasmic membrane) [7]. The precise mechanism of bacitracin resistance by ABC-transporters is not yet fully understood [12].

The expression of most resistance genes is induced in the presence of the respective antibiotic. For example, the *van* operons are induced in the presence of vancomycin by the two-component systems VanS-VanR or VanS_B_-VanR_B_ for VanA- and VanB-type resistance, respectively [11], [13]. Bacitracin-dependent induction of *bcrAB* is mediated by the one-component transmembrane regulator BcrR [7], [14]. While the regulators and target promoters, as well as the conditions leading to induction are known, we lack in-depth understanding of the molecular mechanisms of regulation. For example, while both VanS and VanS_B_ respond to vancomycin, their sensory domains differ considerably in size with 37 amino acids for VanS and 103 residues for VanS_B_, and share only low sequence similarity [15]. It is therefore difficult to envisage the same sensing mechanism for both proteins. It is similarly unclear how BcrR detects bacitracin, because the protein lacks any obvious extracellular domains but is nevertheless able to directly interact with its substrate [14], [16]. Additionally, it is not known how a membrane-bound transcriptional regulator like BcrR activates transcription from its target promoter. While a direct interaction with RNA-polymerase has been proposed [16], experimental evidence is lacking to date.

Sensory perception of antimicrobial substances by bacteria is a first and essential step in antibiotic resistance, and a thorough understanding of the mechanisms involved would provide an important basis for the development of new drugs to combat resistance. However, in many genera, e.g. the enterococci, investigations are hampered by the difficulty to manipulate these bacteria genetically. Although more and more genetic tools are becoming available for enterococci, poor transformability of many strains, including clinical isolates, still impedes studies involving, for example, high-throughput or detailed mutagenic approaches. To circumvent these problems, heterologous hosts have been chosen, often using *E. coli* [17], or electro-transformable laboratory strains of *E. faecalis* [7], [14]. The latter provide improved transformability, but no additional genetic tools, while the former host does not appear well suited to study resistance against cell wall active compounds, due to the major differences between the Gram-positive and Gram-negative cell envelope. Alternatively, *Bacillus subtilis* has been used successfully for the functional expression of the VanS-VanR two-component system of *E. faecalis*, as well as of the VanB-type resistance proteins [1], [18]. Like *E. coli*, *B. subtilis* is easy to manipulate and a large number of genetic tools are available. The G+C contents of *B. subtilis* (43.5%) and of *E. faecalis* (37.5%) are comparable, which is of great advantage for heterologous gene expression. Furthermore, the transcription machinery in both organisms is sufficiently similar to facilitate the interaction of heterologous transcriptional regulators with the native machinery, as has been shown *in vitro* for activation of *B. subtilis* RNA polymerase by *E. faecalis* BcrR [16]. Importantly for the present application, the intrinsic resistance mechanisms of *B. subtilis* against cell wall antibiotics are well understood [19], [20], allowing directed deletion of genes to create a clean genetic background.

In the present study, we have used two well-understood examples from *E. faecalis* to develop and validate *B. subtilis* as a platform for studying the regulatory mechanisms leading to resistance against cell wall-active antibiotics. To test the feasibility of our approach and determine the optimal genetic background of the host, we chose the one-component regulator BcrR and could show full functionality with highly similar behaviour to its native context. This set-up was then applied to the VanS_B_-VanR_B_ two-component system. A previous attempt at heterologous expression of this system in *B. subtilis* had resulted in a constitutively active behaviour [18]. Optimization of expression levels and growth conditions now resulted in vancomycin-dependent induction of the target promoter, further supporting the suitability of *B. subtilis* as host organism.

## Materials and Methods

### Bacterial strains and growth conditions

All strains used in this study are listed in Table 1. *E. coli* DH5αand XL1-blue were used for cloning. *E. coli* and *B. subtilis* were grown routinely in Luria-Bertani (LB) medium at 37°C with agitation (200 rpm). *B. subtilis* was transformed by natural competence as previously described [21]. Selective media contained ampicillin (100 μg ml^−1^ for *E. coli*), chloramphenicol (5 μg ml^−1^ for *B. subtilis*), kanamycin (10 μg ml^−1^ for *B. subtilis*), erythromycin 1 μg ml^−1^ with lincomycin 25 μg ml^−1^ (for macrolide-lincosamide-streptogramin B (mls) resistance in *B. subtilis*) or spectinomycin (100 μg ml^−1^ for *B. subtilis*). Bacitracin was supplied as the Zn^2+^-salt. Unless otherwise stated, media for strains carrying pXT-derived constructs contained 0.2% (w/v) xylose for target gene expression. Solid media contained 1.5% (w/v) agar. Growth was measured as optical density at 600 nm wavelength (OD_600_).

**Table 1:** Plasmids and strains used in this study.

Name	Description^a^	Source
Vectors		
pAC6	Vector for transcriptional promoter fusions to *lacZ* in *B. subtilis*, integrates in *amyE*; cm^r^	[22]
pAH328	Vector for transcriptional promoter fusions to *luxABCDE* in *B. subtilis*; integrates in *sacA*; cm^r^	[23]
pMAD	Vector for construction of unmarked deletions in *B. subtilis*, temperature sensitive replicon; mls^r^	[25]
pXT	Vector for xylose-inducible gene expression in *B. subtilis*; integrates in *thrC*; spc^r^	[24]
Plasmids		
pAMbcr1	*E. coli-E. faecalis* shuttle vector containing a 4.7 kb EcoRI-fragment encompassing the *bcrR-bcrABD* locus of *E. faecalis* AR01/DGVS	[7]
pCF102	pMAD containing the joined “up” and “down” fragments for unmarked deletion of *bceRS-bceAB*	This study
pCF104	pMAD containing the joined “up” and “down” fragments for unmarked deletion of *psdRS-psdAB*	This study
pCF119	pMAD containing the joined “up” and “down” fragments for unmarked deletion of *yxdJK-yxdLM-yxeA*	This study
pCF132	pXT containing the *vanR_B_S_B_* operon of *E. faecalis* V583	This study
pCF133	pAH328 containing P*_vanYB_* of *E. faecalis* V583 from -215 to +65 relative to the *vanY_B_* start codon	This study
pES601	pAC6 containing P*_bcrA_* of *E. faecalis* AR01/DGVS from -219 to +170 relative to the *bcrA* start codon	This study
pES701	pXT containing *bcrR* of *E. faecalis* AR01/DGVS	This study
pES702	pXT containing the *bcrR-bcrAB* region of *E. faecalis* AR01/DGVS	This study
pNTlux101	pAH328 containing P*_bcrA_* of *E. faecalis* AR01/DGVS from -219 to +170 relative to the *bcrA* start codon	This study
*E. coli*		
DH5α	*supE44* Δ*lacU169(*φ*80lacZ*Δ*M15) hsdR17 recA1 endA1 gyrA96 thi-1 relA1*	[39]
XL1-Blue	*recA1 endA1 gyrA96 thi-1 hsdR17 supE44 relA1 lac F′::*Tn*10* *proAB lacI^q^* Δ(*lacZ*)M15]	Stratagene
*E. faecalis*		
AR01/DGVS	Plasmid-cured clinical isolate; bac^r^	[7]
V583	Sequenced clinical strain containing plasmids pTEF1, pTEF2, pTEF3; van^r^	[40]
*B. subtilis*		
W168	Wild-type, *trpC2*	Laboratory stock
SGB34	W168 *thrC*::pES702	This study
SGB35	TMB035 *thrC*::pES702	This study
SGB36	TMB035 *thrC*::pES702 *amyE*::pES601; kan^r^, spc^r^, cm^r^	This study
SGB40	W168 *thrC*::pES701 *amyE*::pES601; spc^r^, cm^r^	This study
SGB42	W168 *thrC*::pES702 *amyE*::pES601; spc^r^, cm^r^	This study
SGB43	TMB035 *thrC*::pES701 *amyE*::pES601; kan^r^, spc^r^, cm^r^	This study
SGB273	TMB1518 *sacA*::pNTlux101; cm^r^	This study
SGB274	TMB1518 *thrC*::pES701 *sacA*::pNTlux101; spc^r^, cm^r^	This study
TMB035	W168 *bceAB*::kan; kan^r^	This study
TMB1518	W168 with unmarked deletions of the *bceRS-bceAB*, *psdRS-psdAB*, *yxdJK-yxdLM-yxeA* loci	This study
TMB1560	TMB1518 *sacA*::pCF133; cm^r^	This study
TMB1562	TMB1518 *thrC*::pCF132 *sacA*::pCF133; spc^r^, cm^r^	This study

a Bac, bacitracin; cm, chloramphenicol; fs, fusidic acid; kan, kanamycin; mls, macrolide-lincosamide-streptogramin B group antibiotics; rif, rifampin; spc, spectinomycin; van, vancomycin; r, resistant.

### Construction of plasmids and genetic techniques

All primer sequences used for this study are listed in Table 2; all plasmid constructs are listed in Table 1.

**Table 2:** Primers used in this study.

Primer name	Sequence (5′-3′)^a^	Use
TM1569	AGTGGATCCTAGGAACGTTTTTACCAAC	*bcrAB* rev
TM1798	TTAAGGATCCGAAAAACCCGTTGATGGACG	*bcrR* fwd
TM1800	TTAAGAATTCTTTTATTTCATTCCCATCTGC	*bcrR* rev
TM1801	TTAAGAATTCTTTTGCTGTTAATCGGCAAG	P*_bcrA_*-*lacZ* fwd
TM1802	TTAAGGATCCCAAGCTGCAACATCATTTTC	P*_bcrA_*-*lacZ* rev
TM2450	AAATTGGATCCGGAAACTACAGACTGTTATG	*vanR_B_* fwd
TM2451	AAATTAAGCTTTATACCTGTCGGTCAAAATC	*vanS_B_* rev
TM2550	AATTTGAATTCTTTGTTCTGGCTGGATTTAC	P*_vanYB_* fwd
TM2551	AATTTACTAGTTCCCCAGATTGTTTCATATG	P*_vanYB_* rev
TM2813	TTAAACTAGTCAAGCTGCAACATCATTTTC	P*_bcrA_*-*lux* rev
TM2347	AATTTGGATCCAGTTTAATATCAACGGCCTG	*yxdJK*-*yxdLM*-*yxeA* deletion up fwd
TM2348	AGGTAATTCTGCAATAGTCC	*yxdJK*-*yxdLM-yxeA* deletion up rev
TM2349	ctattgcagaattacctGGAAGAAGTCAAGTTTGAAG	*yxdJK-yxdLM-yxeA* deletion down fwd
TM2350	AATTTGGATCCTTCTGCTTCCGAAAAAACAG	*yxdJK-yxdLM-yxeA* deletion down rev
TM2351	AATTTGGATCCGAGGAAGCAAAAGGAAATC	*bceRS-bceAB* deletion up fwd
TM2352	CTTGATTTCATGAAACAGCG	*bceRS-bceAB* deletion up rev
TM2355	ctgtttcatgaaatcaagATGGTGTTATATACTGCGC	*bceRS-bceAB* deletion down fwd
TM2356	AATTCCATGGACGAATCCAGTTATCATAGC	*bceRS-bceAB* deletion down rev
TM2357	AATTTGGATCCCTACGATCTAAATGGTTTCC	*psdRS-psdAB* deletion up fwd
TM2358	ATTTTTGAAGATGACCGCCC	*psdRS-psdAB* deletion up rev
TM2361	cggtcatcttcaaaaatGTCATATTTATAAGCGTGCTG	*psdRS-psdAB* deletion down fwd
TM2362	AATTCCATGGAGAGATTGAAGCATTCATCG	*psdRS-psdAB* deletion down rev

a Restriction sites are underlined; overlaps to other primers for PCR fusions are shown by lower case letters.

Transcriptional promoter fusions of P*_bcrA_* to *lacZ* or bacterial luciferase (*luxABCDE*) were constructed in vectors pAC6 [22] or pAH328 [23] by the sites of EcoRI/BamHI and EcoRI/SpeI, respectively, obtaining plasmids pES601and pNTlux101, respectively. The transcriptional promoter fusion of P*_vanYB_* to bacterial luciferase was cloned into the EcoRI and SpeI sites of vector pAH328 creating plasmid pCF133. The exact regions contained in the constructs are given in Table 1.

For heterologous, xylose-inducible expression of *bcrR* or *bcrR-bcrAB* in *B. subtilis* (pES701 and pES702) the respective DNA fragments were amplified from the plasmid pAMbcr1 [7] and cloned in the vector pXT [24] using the BamHI and EcoRI restriction sites, placing the genes under the control of the vector's *xylA*-promoter. Plasmid pCF132 was constructed by inserting *vanR_B_S_B_* from *E. faecalis* V583 into the BamHI and HindIII sites of vector pXT for heterologous, xylose-inducible expression in *B. subtilis*.

Constructs for unmarked gene deletions in *B. subtilis* were cloned into the vector pMAD [25]. For each operon to be deleted, 800–1000 bp fragments located immediately before the start codon of the first gene (“up” fragment) and after the stop codon of the last gene (“down” fragment) were amplified. The primers were designed to create a 17–20 bp overlap between the PCR-products (Table 2), facilitating fusion of the fragments by PCR overlap extension and subsequent cloning into pMAD. Gene deletions were performed as previously described [25].

All constructs were checked for PCR-fidelity by sequencing, and all created strains were verified by PCR using appropriate primers.

### Antimicrobial susceptibility assays

All cultures were grown in Mueller-Hinton (MH) medium for antibiotic susceptibility assays [26]. Minimal inhibitory concentration (MIC) of bacitracin and vancomycin were determined by broth-dilution assays. Freshly grown overnight cultures of *B. subtilis* in MH medium were used as inoculum at a dilution of 1∶500. After 24 h incubation in the presence of two-fold serial dilutions of the antibiotic the MIC was scored as the lowest concentration where no growth was observed.

### β-Galactosidase assays

Cells were inoculated from fresh overnight cultures and grown in LB medium at 37°C with aeration until they reached an OD_600_ between 0.4 and 0.5. The cultures were split into 2 mL aliquots and challenged with different concentrations of bacitracin with one aliquot left untreated. After incubation for an additional 30 min at 37°C with aeration, the cultures were harvested and the cell pellets were frozen at −20°C. β-galactosidase activities were determined as described, with normalization to cell density [27].

### Luciferase assays

Luciferase activities of *B. subtilis* strains were assayed using a Synergy 2 multi-mode microplate reader from BioTek controlled by the software Gen5. LB medium was inoculated 1∶500 from overnight cultures, and each strain was grown in 100 μl volumes in a 96-well plate. Cultures were incubated at 37°C with shaking (intensity: medium), and the OD_600_ was monitored every 10 min. At an OD_600_ of 0.02 (4–5 doublings since inoculation; corresponding to OD_600_ = 0.1 in cuvettes of 1 cm light-path length), either bacitracin was added to final concentrations of 0.03, 0.1, 0.3, 1 μg ml^−1^, or vancomycin to final concentrations of 0.01, 0.025, 0.05, 0.25 μg ml^−1^; in all cases one well was left untreated. Cultures were further incubated for 2 h, and the OD_600_ and luminescence (endpoint-reads; 1 s integration time; sensitivity: 200) were monitored every 5 min. OD_600_ values were corrected using wells containing 100 μl LB medium as blanks. Raw luminescence output (relative luminescence units, RLU) was normalized to cell density by dividing each data-point by its corresponding corrected OD_600_ value (RLU/OD).

## Results and Discussion

### Functional transfer of the BcrR-BcrAB bacitracin resistance system to *B. subtilis*

**Figure 1:**
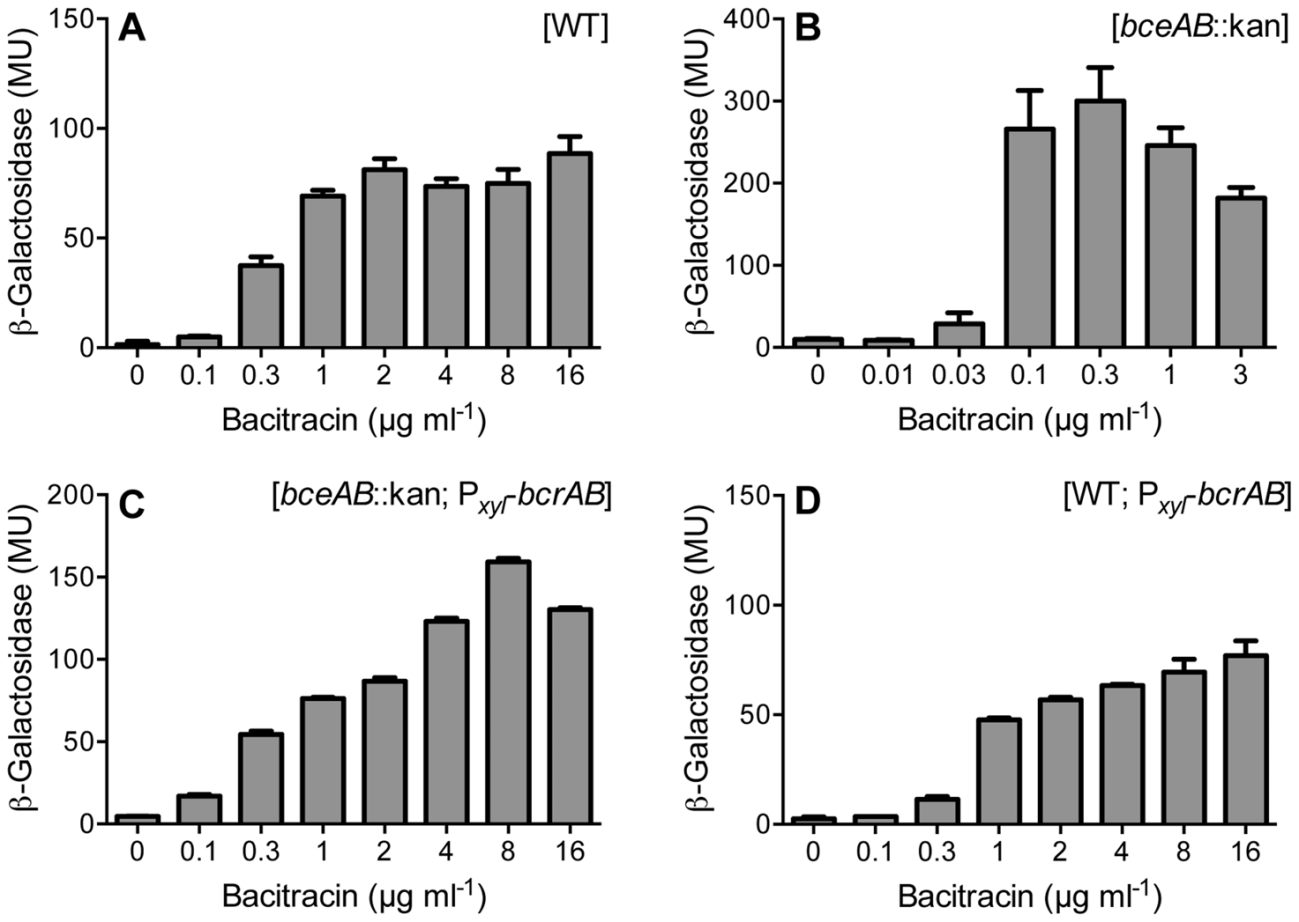
BcrR-dependent induction of P*_bcrA_* by bacitracin in *B. subtilis*. The P*_bcrA_*-*lacZ* reporter construct pES601 was introduced into different strains of *B. subtilis* producing either BcrR or BcrR and BcrAB. The relevant genes for bacitracin transporters in each strain are given at the top right of each graph. (A) SGB40; wild-type (WT) *B. subtilis* with BcrR. (B) SGB43; *bceAB*::kan mutant with BcrR. (C) SGB36; *bceAB*::kan mutant with BcrR and BcrAB. (D) SGB42; wild-type *B. subtilis* with BcrR and BcrAB. Cultures growing exponentially in the presence of 0.2% (w/v) xylose were challenged with different concentrations of bacitracin as indicated for 30 min, and β-galactosidase activities, expressed in Miller Units (MU), were determined. Results are shown as the mean plus standard deviation of three to four biological replicates.

In *E. faecalis*, expression of the genes *bcrAB* that encode the bacitracin resistance transporter BcrAB is controlled solely by the one-component regulator BcrR [14]. This regulator is encoded by a gene directly upstream of the transporter operon, but as an independent transcriptional unit [7]. To test if BcrR could be functionally produced in *B. subtilis*, we introduced a transcriptional fusion of its target promoter, P*_bcrA_*, to *lacZ* (pES601), together with an expression construct of *bcrR* controlled by a xylose-inducible promoter (pES701), into the wild-type strain. Addition of increasing concentrations of bacitracin led to a strong upregulation (approximately 80-fold) of promoter activities with a threshold concentration for induction of 0.3 μg ml^−1^ (Fig. 1A). No promoter activities above background (ca. 1 Miller Unit (MU)) could be detected in a strain lacking BcrR (data not shown), demonstrating that the observed induction was indeed due to BcrR activity. It was shown previously that the sensitivity of BcrR is increased in a strain of *E. faecalis* lacking BcrAB, and this was attributed to competition between the transporter and BcrR in bacitracin binding [14]. While *B. subtilis* itself does not contain a BcrAB-like transporter, it nevertheless possesses a transport system for bacitracin resistance, BceAB, belonging to a different family of transporters [28]. To test if this unrelated transporter could also influence the sensitivity of BcrR, we next introduced the expression and reporter constructs into a strain carrying a *bceAB*::kan deletion (TMB035). Here, the threshold for induction was ten-fold lower at 0.03 μg ml^−1^ bacitracin, with 0.1 μg ml^−1^ leading to full induction. Furthermore, the maximal amplitude of induction was significantly increased (p = 0.006) to more than 200-fold (Fig. 1B). Therefore, the BceAB transporter of *B. subtilis* appeared to decrease the availability of bacitracin for detection by BcrR, similar to the effect of BcrAB in *E. faecalis*.

We next introduced a construct containing *bcrR* under control of the xylose-inducible promoter followed by *bcrAB* under BcrR-dependent control of its native promoter (pES702) into TMB035 (*bceAB*::kan). In this strain, the induction behaviour was comparable to that of wild-type *B. subtilis* carrying BcrR alone (Fig. 1C). Introduction of the same construct into the wild-type background produced a strain harbouring both transporters, BceAB and BcrAB. While the induction threshold was not significantly altered compared to strains possessing only one transporter, the amplitude of induction was lowered to approximately 50-fold (Fig. 1D). These data clearly show that both BceAB and BcrAB are able to compete with BcrR for bacitracin binding and closely reflect the behaviour of the system in *E. faecalis*. As stated above, this competition is most likely due to removal of bacitracin by the transporters.

The decreased sensitivity of P*_bcrA_* induction in strains harbouring the construct of *bcrR* together with *bcrAB*, with the latter being controlled by its native promoter (Fig. 1C and D), further implied that *bcrAB* was expressed in a BcrR-dependent manner in *B. subtilis*. We therefore wanted to test if this construct was also able to impart bacitracin resistance to the *B. subtilis* host. The minimal inhibitory concentration (MIC) of bacitracin was strongly reduced from 128 μg ml^−1^ in the wild-type to 2–4 μg ml^−1^ in the *bceAB*-deleted strain TMB035 (Table 3), consistent with earlier reports [20], [29]. Introduction of the *bcrR*-*bcrAB* construct increased the resistance of the *bceAB*-deleted strain to 32 μg ml^−1^ (Table 3). This degree of protection conferred to *B. subtilis* (i.e. 8- to 16-fold increase in MIC) is the same as that conferred to *E. faecalis* itself, where BcrAB raises the MIC from 32 μg ml^−1^ to >256 μg ml^−1^ [7]. The difference in final resistance reached is due to the differing degrees of intrinsic bacitracin resistance between the two hosts. Additional expression of the *E. faecalis* transporter in wild-type *B. subtilis* could not further increase its resistance (Table 3). In fact we have to date been unable to raise the MIC of the wild-type strain, even with overproduction of its native BceAB transporter (own unpublished observation), suggesting that the level of resistance is not limited by transport capacity.

**Table 3:** Antibiotic susceptibility of *B. subtilis* strains.

Strain	Relevant resistance proteins	Bacitracin MIC^a^ (μg ml^−1^)	Vancomycin MIC^a^ (μg ml^−1^)
W168	BceAB^+^	128	0.25
TMB035	BceAB^−^	2–4	0.25
TMB1518	BceAB^−^	4	0.25
SGB34	BceAB^+^, BcrR-BcrAB^+^	128	0.25
SGB35	BceAB^−^, BcrR-BcrAB^+^	32	0.25

a Minimal inhibitory concentrations (MIC) determined from three biological replicates; where a range of concentrations is given, results varied between replicates.

Taken together, our results demonstrate full functionality of the *E. faecalis* Bcr-system in *B. subtilis*, both regarding gene regulation and bacitracin resistance. Importantly, however, the native resistance determinants of the *B. subtilis* host were shown to interfere with the sensitivity and amplitude of promoter induction and masked the resistance imparted by the introduced system. This observation is addressed in the following section.

### Development of a sensitive recipient strain

When employing a heterologous host for functional studies of resistance and associated regulatory systems, it is of vital importance to consider any potential interference from intrinsic resistance determinants. One advantage of using *B. subtilis* as the heterologous host is that its resistance determinants against cell wall antibiotics are very well known. Several proteins were shown to contribute to broad-spectrum protection from charged antimicrobial peptides, for example by modification of teichoic acids in the cell envelope [30], but most of these mechanisms are not drug-specific. In contrast, antimicrobial peptide transporters such as the BceAB system described above, are thought to function by removal of the antibiotic from its site of action [12], [20], [31]–[33], and are thus likely to interfere with heterologously introduced resistance determinants. *B. subtilis* possesses three paralogous systems of differing substrate specificities: BceAB mediates resistance against bacitracin, mersacidin, actagardine and plectasin [20], [31]; PsdAB confers resistance against a broad-range of lipid II-binding lantibiotics such as nisin or gallidermin [31]; for YxdLM no role in resistance has been identified to date, but it's expression is induced in response to the human cathelicidin LL-37 [34]. All three transporters are encoded together with an operon for a two-component regulatory system, BceRS, PsdRS and YxdJK, respectively, which controls expression of its corresponding transporter operon [28], [31], [35].

**Figure 2:**
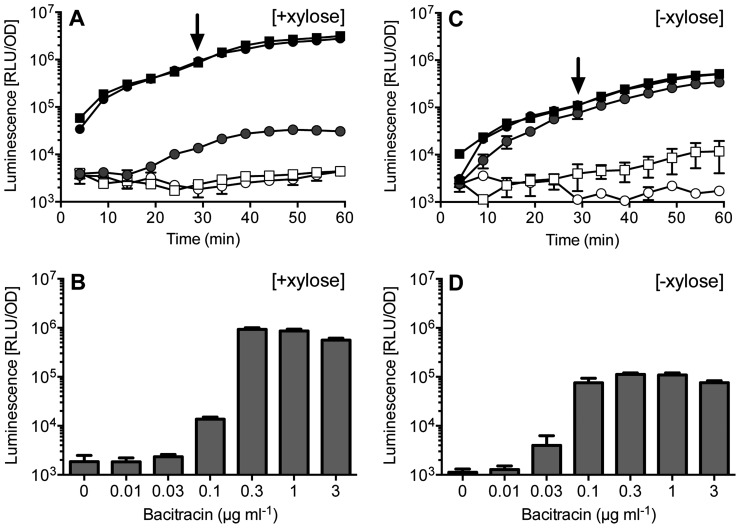
Time-resolved induction of P*_bcrA_* by bacitracin in an unmarked, sensitive *B. subtilis* recipient strain. SGB274, carrying unmarked deletion of *bceRS-bceAB*, *psdRS-psdAB*, *yxdJK-yxdLM-yxeA* and harbouring the P*_bcrA_*-*lux* reporter construct pNTlux101 and *bcrR* expression construct was grown in the presence of 0.2% (w/v) xylose (panels A and B), or in the absence of xylose (panels C and D). In early exponential phase (t = 0 min), bacitracin was added to final concentrations of 0 (open circles) 0.03 μg ml^−1^ (open squares), 0.1 μg ml^−1^ (grey circles), 0.3 μg ml^−1^ (solid circles) or 1 μg ml^−1^ (solid squares), and luminescence normalized to optical density (RLU/OD) was monitored. (A, C) Time-course of promoter induction over 60 min after bacitracin-challenge. (B, D) Dose-response at 30 min post-induction; the time point is labelled with the arrow in the panels above. Results are shown as the mean and standard deviation of three biological replicates.

To obtain a recipient strain that is well suited for the study of resistance mechanisms against cell wall antibiotics from *E. faecalis* and potentially also other genetically intractable Gram-positive bacteria, we therefore created unmarked deletions of all three entire genetic loci, *bceRS*-*bceAB*, *psdRS*-*psdAB* and *yxdJK*-*yxdLM*-*yxeA*. *yxeA* is a small gene of unknown function that may form a transcriptional unit with *yxdLM* and was therefore included in the deletion. To test for the absence of interference, we then introduced the *bcrR* expression construct pES701 used above into the triple deletion strain, TMB1518. While our study was in progress, the Losick-laboratory developed a new reporter system for *B. subtilis*, based on the bacterial luciferase operon *luxABCDE*, which allows time-resolved, semi-automated analyses of transcriptional promoter fusions [23], [36]. To test the applicability of this reporter for our purposes, we inserted the BcrR target promoter P*_bcrA_* upstream of the *lux* operon and introduced this construct into the triple deletion strain harbouring BcrR. At high expression levels of BcrR due to induction by xylose, addition of bacitracin to growing cultures of this strain resulted in a rapid response, with a more than ten-fold increase of promoter activity within 5 min after addition of 1 μg ml^−1^ bacitracin (Fig. 2A). Only background luminescence (ca. 10^3^ relative luminescence units (RLU) per OD) was observed in the absence of bacitracin or in a strain lacking BcrR (Fig. 2A and data not shown). Analysis of promoter activities 30 min post-induction showed a similar dose-response behaviour (Fig. 2B) compared to the corresponding *lacZ* reporter strain shown above (Fig. 1B). While the threshold concentration for induction appeared slightly increased for the P*_bcrA_*-*lux* construct, possibly due to the different growth conditions in 96-well plates compared to test-tubes, the maximal amplitude of induction was approximately doubled to over 500-fold, which can most likely be attributed to the very low background luminescence obtained with luciferase assays. Therefore both the *lacZ* and *lux* reporters are equally suitable to determine dose-response behaviours of regulatory systems, while the *lux* reporter offers higher sensitivity and additionally allows time-resolved analyses for dynamic studies.

To test if the cellular protein levels of a one-component regulator like BcrR affected the promoter induction behaviour, the same experiments were also carried out in the absence of xylose, relying on the basal activities of the P*_xylA_*-promoter for *bcrR* expression (Fig. 2C and D). Under these conditions, the maximal promoter activities were reduced approximately eight-fold (p = 0.0003). Considering that the difference in P*_xylA_* activity in the presence and absence of xylose is ten-fold under the conditions used here [36], this difference in BcrR-activity is likely directly due to a reduced copy number of BcrR in the cell. However, the dose-response behaviour was again similar to previous results, with a threshold concentration for induction in the range of 0.03 to 0.1 μg ml^−1^ bacitracin. Thus the overall function of BcrR was robust to changes in expression, with differences in protein levels merely affecting the amplitude of induction but not the response to the stimulus.

### Qualitative activity assays on solid media for screening applications

**Figure 3:**
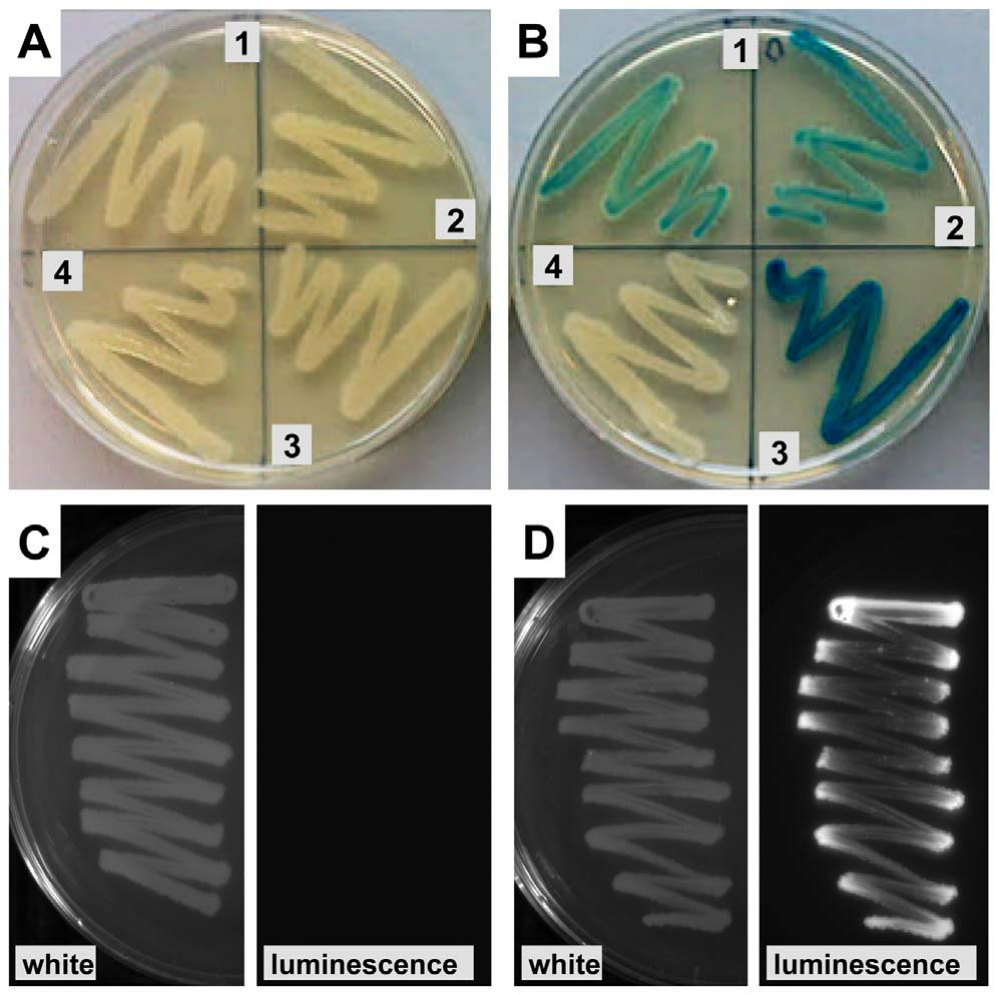
Functionality of the reporter systems on solid media. Strains of *B. subtilis* harbouring the P*_bcrA_*-*lacZ* reporter (A and B) or the P*_bcrA_*-*luxABCDE* reporter (C and D) were grown on agar plates containing 0 μg ml^−1^ (A and C), 0.3 μg ml^−1^ (D) or 1 μg ml^−1^ (B) bacitracin. (A, B) Blue colouration due to reporter induction is depicted by the dark grey shading of bacterial growth. Sector 1, SGB40 (BcrR^+^, BceAB^+^); sector 2, SGB36 (BcrR^+^, BcrAB^+^); sector 3, SGB43 (BcrR^+^); sector 4, SGB42 (BcrR^+^, BceAB^+^, BcrAB^+^). Plates contained 200 μg ml^−1^ X-Gal. (C, D) Plates inoculated with SGB237 (BcrR^+^) were photographed under white light (left sub-panels), followed by detection of luminescence in the dark (right sub-panels); the same sector of the agar plates is shown in both sub-panels.

To elucidate the molecular mechanisms of stimulus perception and signal transduction in regulatory systems, random or site-directed mutagenesis is often used. Particularly in the case of random mutagenesis approaches, but also for (synthetic) DNA-libraries, assays performed on solid media greatly facilitate screening of large numbers of clones. To evaluate the *lacZ* and *lux* reporters for such applications, the derived BcrR/P*_bcrA_* reporter strains were streaked onto agar plates in the absence or presence of bacitracin. Strains harbouring the P*_bcrA_*-*lacZ* fusions showed a blue colouration on XGal-containing agar plates in the presence of inducing concentrations of bacitracin, but remained white in its absence (Fig. 3A and B). As observed before in the quantitative assays, presence of the transporters BceAB or BcrAB diminished the intensity of colouration (Fig. 3B, sectors 1 and 2). In the strain possessing both transporters, bacitracin concentrations of at least 10 μg ml^−1^ were required to produce blue colonies (data not shown), consistent with the low promoter activities reported above for this strain. The reporter strain harbouring BcrR and the P*_bcrA_*-*lux* construct showed strong luminescence when grown on agar plates containing 0.3 μg ml^−1^ bacitracin, and no detectable luminescence in its absence (Fig. 3C and D).

Both reporter constructs are therefore suitable for screening libraries of clones for promoter induction and are applicable for high-throughput approaches. In principle, screens for loss-of-function as well as gain-of-function mutations can be performed, depending on experimental design. This set-up offers a great advantage over studies performed directly in *E. faecalis*, where it is much more difficult to obtain large numbers of transformants than in the naturally competent *B. subtilis*. Importantly, the output of both promoters is sufficiently sensitive to allow assays to be performed at sub-lethal concentrations of the antibiotic, at least in the case of the Bcr-system. The feasibility of this approach was recently demonstrated in a study that identified essential residues in the *B. subtilis* bacitracin resistance transporter BceAB [29], and the same strategy should be applicable to the heterologous set-up described here.

### Functional transfer of the VanS_B_-VanR_B_ two-component system to *B. subtilis*

**Figure 4:**
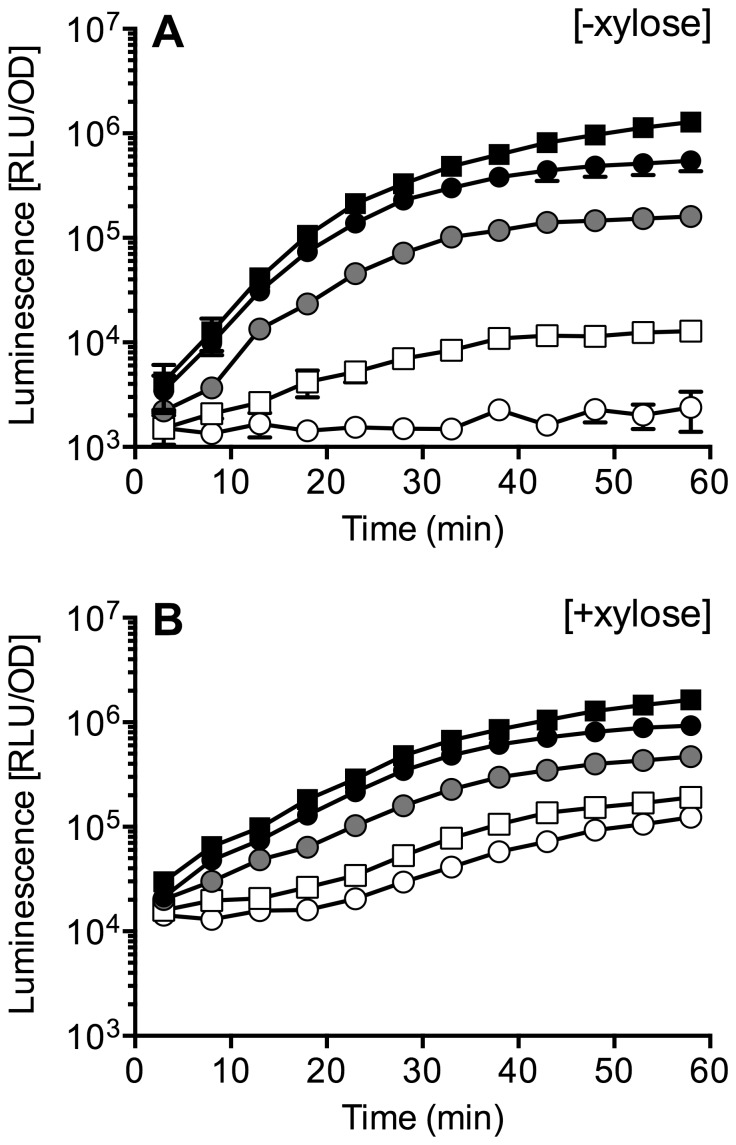
VanS_B_-VanR_B_-dependent induction of P*_vanYB_* by vancomycin in *B. subtilis*. Both the P*_vanYB_*-*lux* reporter construct pCF133 and the P*_xylA_*-*vanR_B_S_B_* expression construct pCF132 were introduced into *B. subtilis* strain TMB1518 (unmarked deletion of *bceRS-bceAB*, *psdRS-psdAB*, *yxdJK-yxdLM-yxeA*). Cultures growing exponentially either (A) in the absence of xylose or (B) in the presence of 0.2% (w/v) xylose were challenged at t = 0 min with 0.01 μg ml^−1^ (open squares), 0.025 μg ml^−1^ (grey circles), 0.05 μg ml^−1^ (solid circles), 0.25 μg ml^−1^ (solid squares) vancomycin, or left untreated (open circles). Luminescence normalized to optical density (RLU/OD) was monitored over 60 min. Results are shown as the mean and standard deviation of three biological replicates.

Following successful transfer of the Bcr-system of *E. faecalis* to *B. subtilis*, we next wanted to test if our set-up could be applied to other regulatory systems. The two-component system VanS-VanR regulating VanA-type vancomycin resistance had previously been shown to be functional in *B. subtilis* [1]. However, heterologous expression of *vanR_B_vanS_B_* encoding the regulatory system for VanB-type resistance had resulted in constitutive expression of the target promoter, P*_vanYB_*, and the authors could show that this was due to constitutive activity of the sensor kinase VanS_B_ under the conditions chosen [18]. To test if vancomycin-dependent modulation of VanS_B_ activity could be obtained by optimization of conditions, we introduced an expression construct of the *vanR_B_vanS_B_* operon under control of the xylose-inducible promoter P*_xylA_* into TMB1518. The activity of the two-component system was monitored as activation of a P*_vanYB_*-*luxABCDE* transcriptional fusion. In the absence of xylose, only low levels of the two-component systems will be produced in the cell, due to basal promoter activity of P*_xylA_*. Under these conditions, addition of increasing concentrations of vancomycin to growing cultures of the reporter strain led to a gradual up-regulation of promoter activity (Fig. 4A). Importantly, and in contrast to previous data, only background activity was observed in the absence of vancomycin (Fig. 4A, open circles). The threshold concentration for induction was 0.01 μg ml^−1^, and a maximum induction of ca. 500-fold was observed in the presence of 0.05–0.25 μg ml^−1^ vancomycin. The MIC of *B. subtilis* for vancomycin was determined as 0.25 μg ml^−1^ for both the wild-type and TMB1518 (Table 3), and therefore higher concentrations were not tested. In the previous study, promoter activities were analysed only in the presence of xylose to ensure high expression levels of the two-component system [18], which may have led to the high basal activities observed. We therefore next repeated the induction experiments, but in the presence of 0.2% xylose, and indeed found ten-fold increased promoter activities in the absence of vancomycin (Fig. 4B). Vancomycin-dependent induction was still observed, but only to a maximum of ten-fold over the uninduced control, due to the higher basal activity.

Together with previously published reports [1], [18], our data show that the regulators of vancomycin resistance in *E. faecalis* can be functionally produced in *B. subtilis*, although the expression levels have to be adjusted for optimal signal-to-background ratios. The full functionality of the VanRS two-component systems, both of VanA-type resistance described previously [1] and VanB-type resistance shown here, validates the biological relevance of the heterologous set-up and paves the way for detailed mechanistic investigations into the respective modes of vancomycin detection. The high degree of competence of *B. subtilis*, for example, allows high-throughput screening of random mutants, synthetic DNA libraries, or chimeric protein fusions, which may lead to discovery of ligand binding sites and thus to elucidation of sensory mechanisms. Promising results can then be validated in a more targeted fashion in *E. faecalis*.

Additionally, Bisicchia and colleagues had reported that vancomycin resistance could be imparted on *B. subtilis* by expression of the VanB-type resistance operon *vanY_B_WH_B_BX_B_*, further extending the applicability of this host organism.

## Conclusions

In summary we here show that *B. subtilis* is well suited to the use as a host for functional production of regulatory systems that control resistance against cell wall active compounds in *E. faecalis*. Our data also show that care has to be taken regarding the genetic background of the host strain and that appropriate expression levels of the regulator genes have to be experimentally determined. Due to the availability of a range of inducible and constitutive promoters, for which strength and dynamic behaviour are very well characterized [36], *B. subtilis* offers a vast potential for optimization of expression levels, again supporting its suitability as a versatile heterologous host. Full functionality of any newly introduced system should of course be validated by comparison of its behaviour between *B. subtilis* and the native host before detailed mechanistic investigations are commenced.

To minimize interference from intrinsic resistance determinants against antimicrobial peptides, we have constructed a *B. subtilis* strain devoid of the most efficient systems. This strain should provide a clean genetic background for the study of a broad range of resistance mechanisms against cell wall active substances, particularly regarding their regulation. In addition to one-component regulation of bacitracin resistance and two-component regulation of vancomycin resistance implemented here, we have successfully applied this set-up to the functional reconstitution of a more complex regulatory and resistance network [37]. It should be noted that the response of *B. subtilis* to antibiotics in general is among the best understood of all bacteria investigated to date [38]. This plethora of available data therefore constitutes an ideal basis for construction of new sensitive recipient strains adapted to the study of resistance and regulatory systems also for other classes of antimicrobials.

Further, we showed that the two reporters, *lacZ* and *luxABCDE*, can both be used for qualitative (high-throughput) screening approaches, for example of mutant libraries, as well as for the quantitative characterization of regulators. Complementation studies with random or directed mutations can thus be initiated in the genetically accessible, highly competent host *B. subtilis*, and promising results then validated directly in *E. faealis*. Construction of the desired heterologous strains will be further aided by a recently established and fully validated tool-box of vectors, promoters, reporters and epitope-tags for engineering of *B. subtilis* [36]. We therefore envisage that the system developed here will aid investigations into the molecular mechanisms of sensory perception of antimicrobials and subsequent signal transduction, the first essential step of antibiotic resistance. Furthermore, this set-up should also be applicable to the study of unrelated resistance systems or even regulatory cascades of diverse functions from other genetically intractable Gram-positive bacteria.
